# Tofacitinib-Induced Steroid-Free Remission in Subcutaneous Sarcoidosis With Neurologic Involvement: A Case Report

**DOI:** 10.7759/cureus.91580

**Published:** 2025-09-04

**Authors:** Dan Hong, Jianchi Ma, Zhenrui Shi

**Affiliations:** 1 Department of Dermatology, Sun Yat-sen Memorial Hospital, Sun Yat-sen University, Guangzhou, CHN; 2 Department of Dermatology, The Seventh Affiliated Hospital, Sun Yat-sen University, Shenzhen, CHN

**Keywords:** janus kinase inhibitors, neurologic sarcoidosis, sarcoidosis, sarcoidosis treatment, subcutaneous sarcoidosis

## Abstract

We report the case of a 62-year-old woman with subcutaneous sarcoidosis and probable neurologic involvement who achieved complete remission with tofacitinib without the use of systemic corticosteroids. The patient presented with multiple firm subcutaneous nodules on the extremities and limb weakness. Skin biopsy demonstrated non-caseating granulomas, and electromyography revealed neurogenic changes consistent with C5-C6 nerve root involvement. Treatment with tofacitinib 5 mg twice daily in combination with methotrexate 7.5 mg weekly led to complete resolution of both cutaneous lesions and neurologic symptoms within two months. Tofacitinib was discontinued after eight months, and remission was sustained at 11-month follow-up. This case illustrates the potential of JAK inhibition as an effective steroid-sparing therapeutic strategy in the management of multisystem sarcoidosis.

## Introduction

Sarcoidosis is a multisystem granulomatous disease that most commonly affects the lungs and lymphatic system, but can also involve the skin and nervous system. Although cutaneous manifestations occur in up to 25% of patients [1], subcutaneous nodules (Darier-Roussy sarcoidosis) are a relatively uncommon variant and frequently associated with systemic disease [2]. Neurologic involvement, or neurosarcoidosis, occurs in roughly 5-10% of sarcoidosis patients and can involve the central and peripheral nervous system with potentially serious morbidity [1,3]. Historically, the first-line treatment for both systemic and neurologic sarcoidosis has been high-dose corticosteroids, often with steroid-sparing immunosuppressants like methotrexate or azathioprine for chronic management. However, prolonged steroid use carries substantial side effects, and some cases are refractory to conventional therapies [4].

## Case presentation

A 62-year-old woman presented with a six-month history of progressive, asymptomatic subcutaneous nodules on her extremities. Three months prior to dermatologic evaluation, the patient had developed an insidious onset of limb weakness, noted as difficulties lifting arms overhead, climbing stairs, squatting, and rising from a seated position. There were no cranial nerve symptoms or bladder/bowel disturbance. Laboratory evaluation showed mildly elevated lactate dehydrogenase (LDH), with normal creatine kinase (CK) and myoglobin (Table 1). Electromyography demonstrated bilateral biceps neurogenic damage, consistent with C5-6 anterior horn or nerve root involvement (Table 2). A cervical spine magnetic resonance imaging (MRI) was considered to evaluate for structural causes, but the patient deferred at that time. An empirical course of neurotrophic B-vitamin therapy was started by the neurologist, but her weakness continued to progress over the ensuing weeks. The patient had a history of hypertension and type 2 diabetes mellitus for several years. She had been taking extended-release nifedipine and glimepiride regularly, with satisfactory control of blood pressure and blood glucose levels.

**Table 1:** Key laboratory findings of the patient.

Parameter	Measured value	Reference range
Lactate dehydrogenase (LDH)	306 U/L	108 - 252 U/L
Creatine kinase (CK)	11 U/L	40 - 200 U/L
Myoglobin	21.4 μg/L	25.0 - 58.0 μg/L
Erythrocyte sedimentation rate (ESR)	105 mm/1^st^ hour	20 mm/1^st^ hour
C-reactive protein (CRP)	20.90 mg/L	< 5 mg/L
Myositis panel	Negative	Negative
Antinuclear antibodies (ANA) test by indirect immunofluorescence (IIF)	< 1:80	< 1:80
ANA panel	Negative	Negative
Quantitative hepatitis B surface antigen (HBsAg)	< 0.05 IU/ml	< 0.08 IU/ml
Quantitative hepatitis B surface antibody (HBsAb)	241.82 IU/L	< 10 or ≥ 10 IU/L
Quantitative hepatitis B envelope antigen (HBeAg)	0.17 COI	< 1.00 COI
Quantitative hepatitis B envelope antibody (HBeAb)	2.24 COI	> 1.00 COI
Quantitative hepatitis B core antibody (HBcAb)	1.76 COI	> 1.00 COI
Carcinoembryonic antigen (CEA)	3.8 ng/ml	≤ 5.0 ng/ml
Alpha-fetoprotein (AFP)	0.99 ng/ml	≤ 7 ng/ml

**Table 2:** Bilateral biceps brachii electroneuromyography abnormality summary.

Parameter	Right	Left	Normal range
Motor unit action potential (MUAP) duration (ms)	14.6	15.3	8-13.7
MUAP amplitude (μV)	780	1363	200-1200

**Figure 1:**
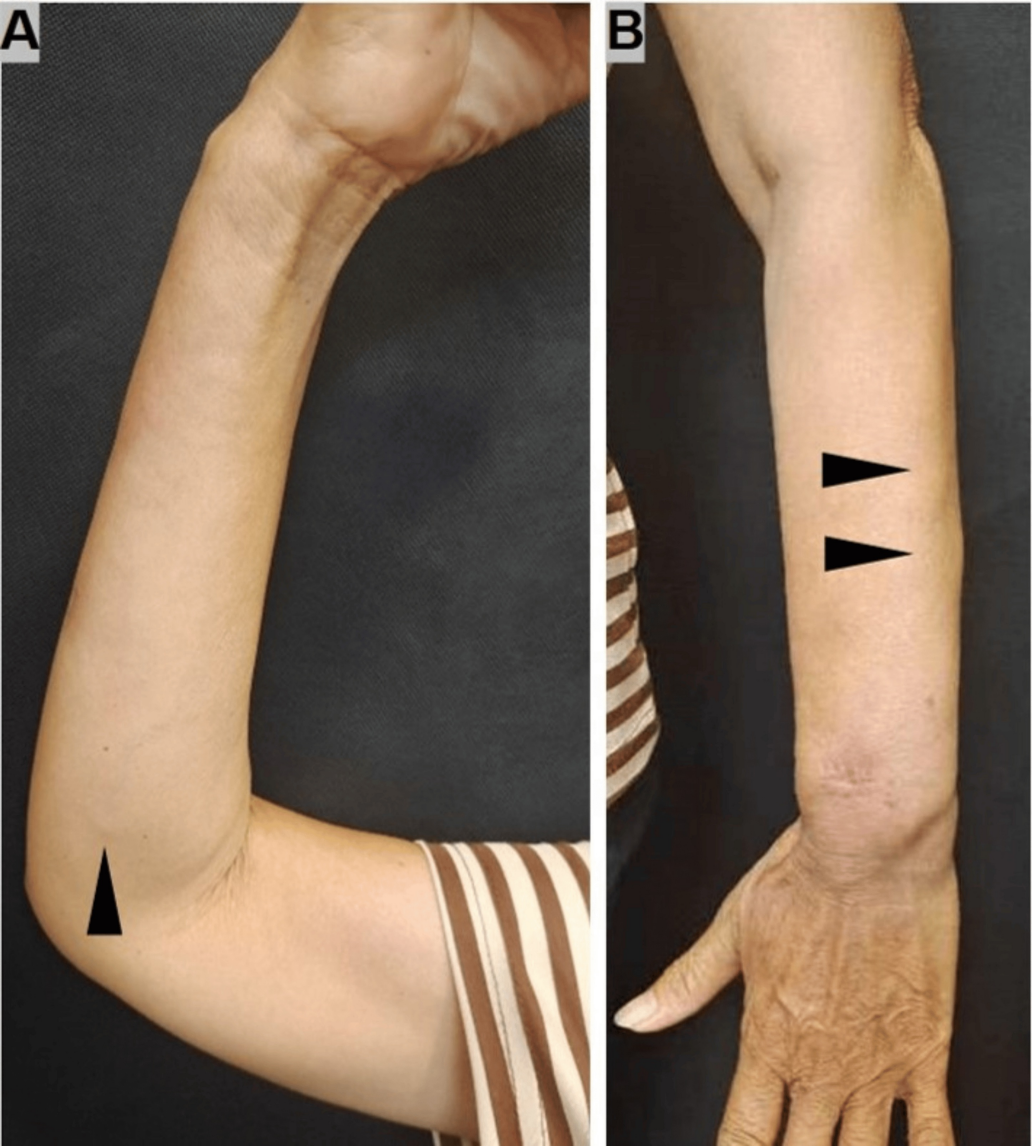
Subcutaneous nodules on the forearms before treatment (Right, A; Left, B).

**Figure 2:**
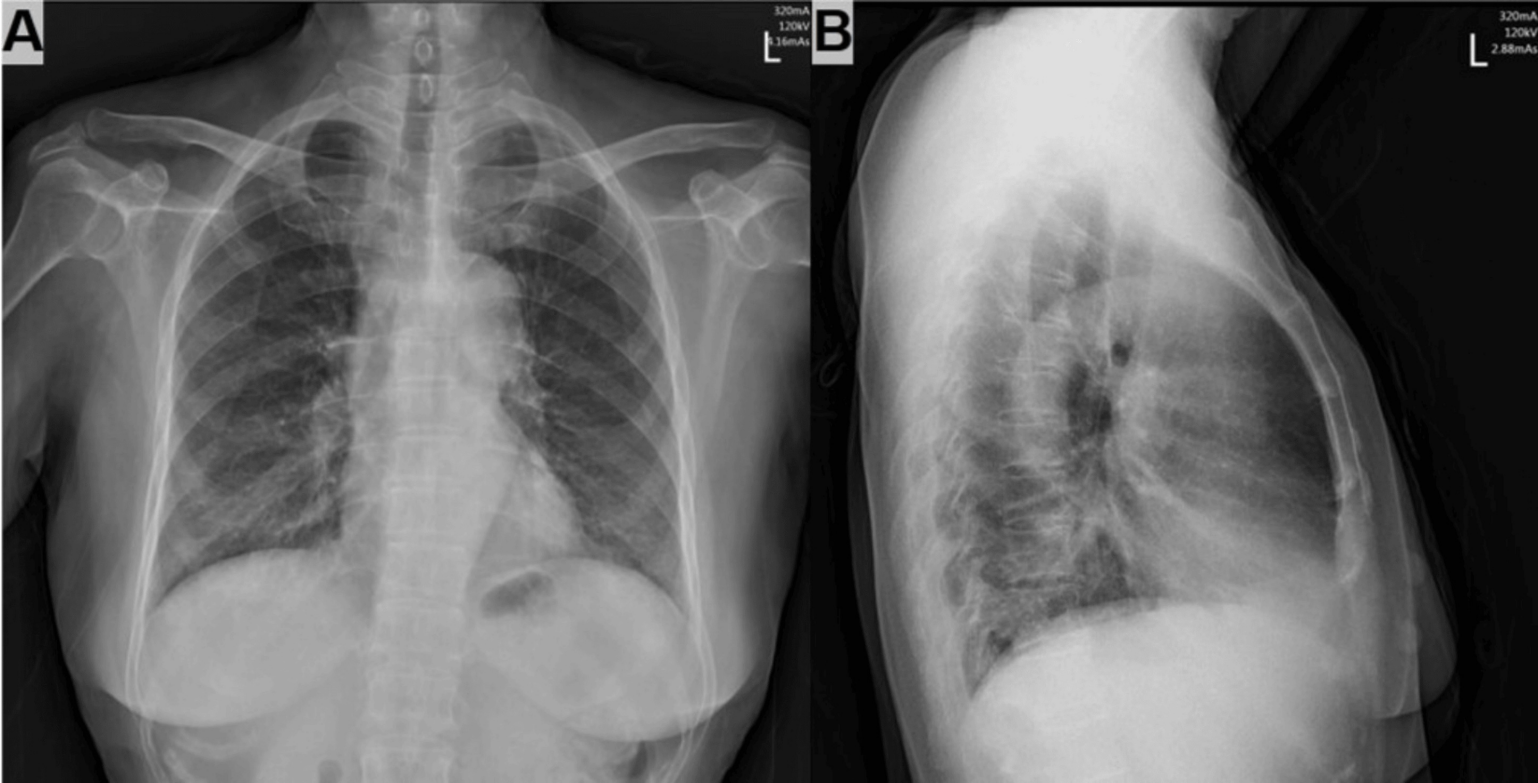
Chest X-ray (posteroanterior view (A) and lateral view (B)) showing mild bilateral interstitial changes.

**Figure 3:**
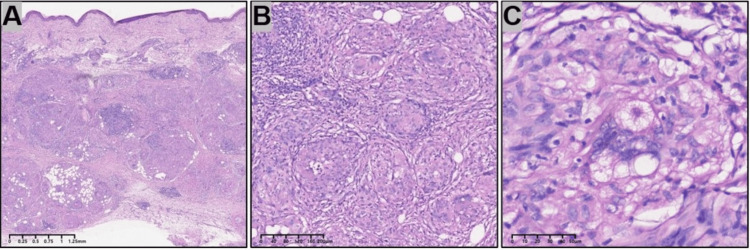
Histopathology (hematoxylin-eosin stain; original magnifications: ×1.5 (A) and ×10 (B)) showing non-caseating granulomas in the dermis composed of epithelioid histiocytes, multinucleated giant cells and occasional asteroid bodies (C).

On examination, multiple firm, non-tender nodules were noted on the forearms and thighs, ranging from 0.5 to 2 cm, without overlying erythema or ulceration (Figure 1). No lymphadenopathy was appreciated. Chest X-ray (Figure 2) showed mild bilateral interstitial changes. Autoimmune antibody profile, tumor markers, and infectious serologies were negative (Table 2). A skin biopsy from the forearm revealed well-formed, non-caseating epithelioid granulomas in the dermis with occasional multinucleated giant cells and scant lymphocytic infiltrate (Figure 3). Periodic acid Schiff plus diastase (PAS-D) and acid-fast stains were negative.

**Figure 4:**
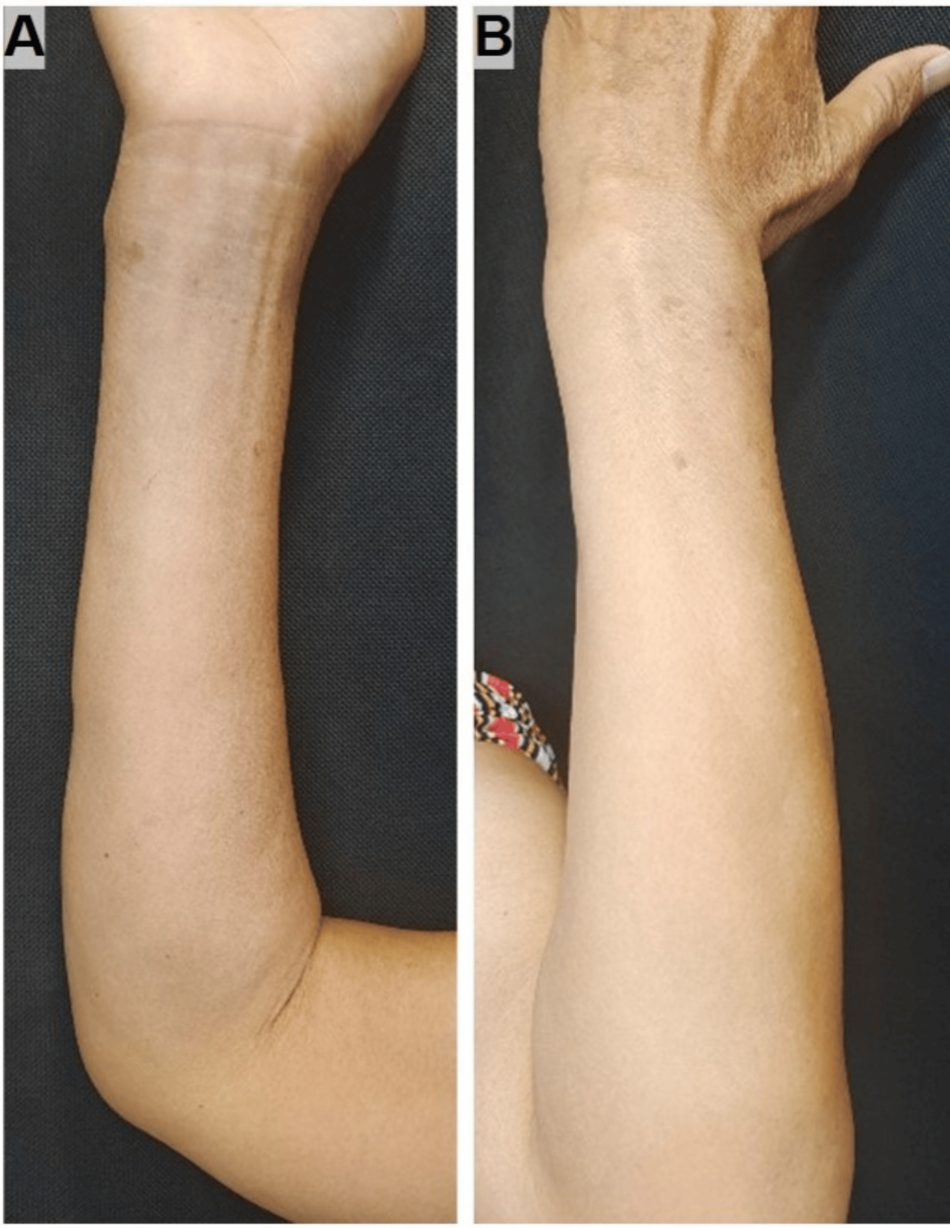
Bilateral forearms at two months post-treatment, showing regression of subcutaneous nodules and normalization of contour (Right, A; Left, B).

A diagnosis of subcutaneous sarcoidosis with suspected neurologic involvement was made. The patient declined corticosteroids due to concerns about metabolic complications. We initiated tofacitinib 5 mg twice daily with methotrexate 7.5 mg weekly. After two months, the nodules had regressed significantly (Figure 4), and the patient reported resolution of limb weakness. After eight months of therapy, the patient’s cutaneous lesions had almost completely resolved and her neurological function was back to baseline. During treatment, the patient remained infection-free in the respiratory, gastrointestinal, and other systems, with normal results on serial monitoring of complete blood count, liver function, and coagulation profiles. A repeat electrophysiological study at eight months showed normalization. At this point, tofacitinib was tapered and discontinued, while methotrexate was reduced to 5 mg weekly as maintenance. At 13 months, the patient remained in complete remission.

## Discussion

This case highlights the potential for Janus kinase (JAK) inhibition to induce steroid-free remission in multisystem sarcoidosis, including suspected neurosarcoidosis. The cutaneous lesions resolved rapidly and completely, and neurologic symptoms improved in parallel, consistent with systemic inflammatory control.

Sarcoidosis granulomas are driven by interferon (IFN)-γ, interleukin (IL)-6, and other cytokines that activate the JAK-signal transducers and activators of transcription (STAT) pathway [5,6]. Tofacitinib inhibits JAK1 and JAK3, disrupting these inflammatory signals. Damsky et al. first reported resolution of cutaneous sarcoidosis using tofacitinib in 2018 [7], and subsequent studies confirmed its efficacy in multiorgan disease [8-10].

In a 2022 open-label trial, tofacitinib induced significant improvement in 10 patients with refractory sarcoidosis, including histologic resolution [5]. A separate study of pulmonary sarcoidosis patients showed that 60% were able to reduce corticosteroids by ≥50% within 16 weeks on tofacitinib, with stable pulmonary function [11].

This patient’s complete remission without corticosteroids, sustained after withdrawal of tofacitinib, suggests durable immune modulation. Notably, neurologic involvement has been underreported in JAK inhibitor studies, making this a valuable addition to the literature. Taken together with prior case reports, our case adds to the growing evidence that JAK-STAT pathway inhibition can induce and maintain sarcoidosis remission across organ systems while minimizing reliance on systemic steroids. However, due to the absence of cervical spine MRI and cerebrospinal fluid analysis, other causes of the neurological symptoms could not be fully ruled out, leaving neurosarcoidosis as a suspected diagnosis. Moreover, the period of observation after treatment was relatively short, necessitating continued patient monitoring. Larger, controlled studies are warranted to further establish the safety and efficacy of JAK inhibitors in sarcoidosis with multiple organ damage.

## Conclusions

This case report demonstrates the successful use of tofacitinib in combination with low-dose methotrexate to achieve complete steroid-free remission in a patient with multisystem sarcoidosis. The rapid and durable response, particularly notable in the context of probable neurologic involvement, underscores the potential of JAK inhibition as an effective steroid-sparing strategy for systemic sarcoidosis, including challenging neurologic manifestations. While promising, larger controlled trials are essential to definitively establish the efficacy, optimal dosing, and long-term safety of JAK inhibitors across the spectrum of sarcoid organ involvement.
